# Redirection of pyruvate flux toward desired metabolic pathways through substrate channeling between pyruvate kinase and pyruvate-converting enzymes in *Saccharomyces cerevisiae*

**DOI:** 10.1038/srep24145

**Published:** 2016-04-07

**Authors:** Sujin Kim, Sang-Jeong Bae, Ji-Sook Hahn

**Affiliations:** 1 School of Chemical and Biological Engineering, Seoul National University, Institute of Chemical Processes, 1 Gwanak-ro, Gwanak-gu, Seoul 08826, Republic of Korea

## Abstract

Spatial organization of metabolic enzymes allows substrate channeling, which accelerates processing of intermediates. Here, we investigated the effect of substrate channeling on the flux partitioning at a metabolic branch point, focusing on pyruvate metabolism in *Saccharomyces cerevisiae*. As a platform strain for the channeling of pyruvate flux, PYK1-Coh-Myc strain was constructed in which *PYK1* gene encoding pyruvate kinase is tagged with cohesin domain. By using high-affinity cohesin-dockerin interaction, the pyruvate-forming enzyme Pyk1 was tethered to heterologous pyruvate-converting enzymes, lactate dehydrogenase and α-acetolactate synthase, to produce lactic acid and 2,3-butanediol, respectively. Pyruvate flux was successfully redirected toward desired pathways, with a concomitant decrease in ethanol production even without genetic attenuation of the ethanol-producing pathway. This pyruvate channeling strategy led to an improvement of 2,3-butanediol production by 38%, while showing a limitation in improving lactic acid production due to a reduced activity of lactate dehydrogenase by dockerin tagging.

In recent decades, metabolic engineering of microbial cells has received a great attention for the production of chemicals, fuels, and pharmaceuticals^1^,^2^,^3^. Amplification of the metabolic pathway toward desired compound and inhibition of the competing pathways are generally adopted engineering strategies to achieve high titer and yield of production. To this end, various methods of regulating gene expression levels and gene knockout/down systems have been applied to metabolic engineering^2^,^4^,^5^,^6^.

On the other hand, several efforts have been focused on the spatial organization of metabolic enzymes, leading to a substrate channeling effect, which has several potential advantages such as preventing the loss of intermediates to competing pathways, reducing the accumulation of toxic intermediates, and protecting unstable intermediates, as well as improving conversion rates^7^,^8^,^9^. Spatial proximity of the pathway enzymes can be achieved by several strategies such as making a fusion protein or a scaffold-based enzyme complex, and localization of the enzymes to subcellular compartments or organelles^4^,^10^. For example, expression of a fusion protein of 4-coumaroyl-CoA ligase and stilbene synthase in *Saccharomyces cerevisiae* resulted in up to 15-fold increase in resveratrol production compared to co-expression of these enzymes^11^. Synthetic protein scaffold was applied to the heterologous mevalonate pathway in *Escherichia coli*, resulting in a 77-fold increase in mevalonate titer with the optimized scaffold architecture^9^. In addition to protein scaffold, RNA- and DNA-based scaffold systems have been constructed and used to produce various target products^12^,^13^,^14^. The strategy of compartmentalization of metabolic pathways to specific organelles, especially mitochondria, using N-terminal targeting sequences, has also been successfully applied to the production of isobutanol, itaconic acid, and acetoin in *S. cerevisiae, Aspergillus niger*, and *Candida glabrata*, respectively^15^,^16^,^17^.

The feasibility of the high-affinity cohesin-dockerin interaction, found in cellulosome complex, has been demonstrated in various applications such as protein purification and enzyme immobilization^18^,^19^,^20^. Previously, three enzymes converting pyruvate to 2,3-butanediol, including α-acetolactate synthase (AlsS) and α-acetolactate decarboxylase (AlsD) from *Bacillus subtilis* and endogenous 2,3-butanediol dehydrogenase (Bdh1), were expressed as dockerin-fused proteins, and then assembled onto a scaffold containing multiple cohesin domains^21^. Although such a substrate channeling strategy was effective in channeling α-acetolactate produced by AlsS into 2,3-butanediol production pathway, it was not efficient to compete with the native ethanol production pathway for pyruvate availability. In this study, we aimed to test the effect of substrate channeling on the flux partitioning of pyruvate, an important metabolite at a metabolic branch point. In *S. cerevisiae*, pyruvate is mainly converted to ethanol in the presence of fermentable carbon sources such as glucose. Pyruvate kinase, which catalyzes the conversion of phosphoenolpyruvate (PEP) to pyruvate, is the enzyme for the last step of glycolysis. By tethering heterologous pyruvate-converting enzymes to pyruvate kinase (Pyk1) using cohesin-dockerin interaction, pyruvate flux was successfully redirected to desired pathways producing lactic acid or 2,3-butanediol.

## Results

### Construction of PYK1-Coh-Myc strain as a platform strain for the channeling of pyruvate flux

**Figure 1:**
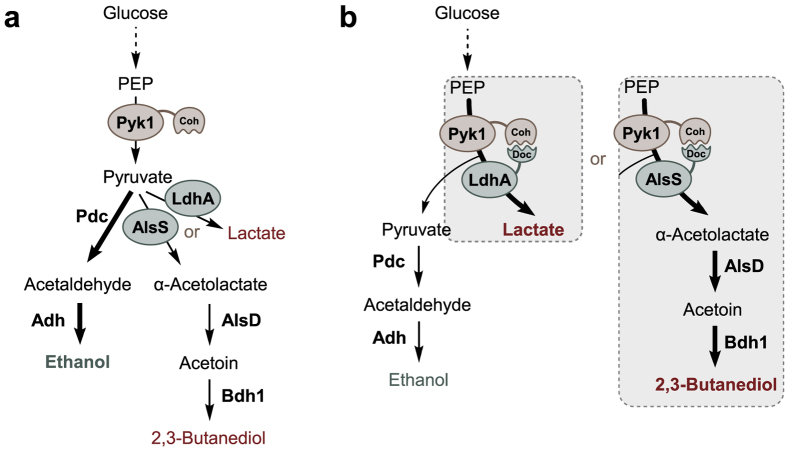
Schematic diagram of the overall concept of this research. Pyruvate kinase (Pyk1) catalyzes the conversion of phosphoenolpyruvate (PEP) to pyruvate, the last step of glycolysis. Pyruvate is mainly converted to ethanol by the sequential actions of pyruvate decarboxylase (Pdc) and alcohol dehydrogenase (Adh) in *S. cerevisiae*. The PYK1-Coh-Myc strain expressing cohesin-tagged Pyk1 was constructed as a platform strain for the pyruvate channeling. (**a**) Overexpression of LdhA for lactate production or AlsS, AlsD, and Bdh1 for 2,3-butanediol production. Because cohesin-tagged Pyk1 cannot interact with native LdhA or AlsS, LdhA or AlsS competes with Pdc for the utilization of pyruvate, the common substrate. (**b**) Overexpression of LdhA-Doc for lactate production or AlsS-Doc, AlsD, and Bdh1 for 2,3-butanediol production. In PYK1-Myc-Coh strain, cohesin-dockerin interaction provides spatial proximity between cohesin-tagged Pyk1 and the dockerin-tagged LdhA or AlsS, resulting in pyruvate channeling to the interacting enzyme.

Pyruvate is a key metabolite of the central metabolism and serves as an important precursor for biosynthesis of several products such as carboxylic acid, alcohols, and amino acids. In *S. cerevisiae*, pyruvate produced by glycolysis is mainly converted to ethanol via acetaldehyde during fermentative growth (Fig. 1). Therefore, if pyruvate serves as a precursor for the production of target products, as in the case of producing lactic acid or 2,3-butanediol, it is very important to redirect the carbon flux from ethanol toward desired target products.

In this study, high-affinity cohesin-dockerin interaction was applied to recruit pyruvate-converting enzymes such as lactate dehydrogenase (LdhA) or α-acetolactate synthase (AlsS) to a pyruvate-forming enzyme, Pyk1 (Fig. 1). Although *S. cerevisiae* has another gene encoding pyruvate kinase, *PYK2*, Pyk1 is known as a main pyruvate kinase^22^. As a platform strain, we generated PYK1-Coh-Myc strain in which *PYK1* gene in the chromosome is C-terminally tagged with cohesin domain and Myc epitope. As a control strain, PYK1-Myc strain where *PYK1* is tagged only with Myc was also constructed.

### Identification of the interaction between cohesin-fused Pyk1 and dockerin-fused enzyme by co-immunoprecipitation

**Figure 2:**
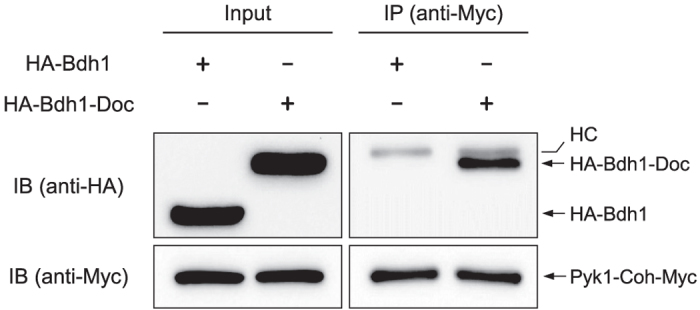
Co-immunoprecipitation (co-IP) between Pyk1-Coh-Myc and dockerin-tagged enzyme. Myc-tagged Pyk1 (Pyk1-Coh-Myc) was immunoprecipitated with anti-Myc antibody from PYK1-Coh-Myc strains expressing either HA-Bdh1 or HA-Bdh1-Doc. Cell lysates before immunoprecipitation (Input) and immunoprecipitates (IP) were analyzed by SDS-PAGE and immunoblotting (IB) with the indicated antibodies. HC indicates the position of the heavy chain of the anti-Myc antibody used for immunoprecipitation.

To ensure the interaction between cohesin and dockerin domains, fused to Pyk1 and a pyruvate-converting enzyme, respectively, we performed co-immunoprecipitation (co-IP) assays using 2,3-butanediol dehydrogenase (Bdh1) as a testing interacting partner of Pyk1. PYK1-Coh-Myc strain was transformed with p415GPD-HA-Bdh1-Doc or p415GPD-HA-Bdh1, expressing N-terminal HA-tagged Bdh1 (HA-Bdh1) with or without C-terminal dockerin domain (Doc). As shown in Fig. 2, HA-Bdh1 with a C-terminal dockerin domain (HA-Bdh1-Doc) was successfully co-precipitated with Pyk1-Coh-Myc, while no interaction was detected between HA-Bdh1 and Pyk1-Coh-Myc protein. These results demonstrate that the dockerin domain of HA-Bdh1-Doc interacts specifically with the cohesin domain of Pyk1. Therefore, this approach connecting Pyk1 to pyruvate-converting enzymes based on cohesin-dockerin interaction could be applied for channeling of pyruvate produced by Pyk1 toward desired products.

### Lactate production using the pyruvate channeling system

In order to test whether co-localization of Pyk1 and pyruvate-converting enzymes through cohesin-dockerin interaction could bring to metabolic channeling of pyruvate to desired metabolic pathways, we first adopted this system to lactate production in *S. cerevisiae*. Lactic acid is a valuable chemical that can be used as a starting material for biodegradable polymers and as an acidulant and preservative in food industry. Although *S. cerevisiae* cannot produce lactate naturally, due to their robust characteristics such as high acid tolerance, there have been many attempts to engineer *S. cerevisiae* for lactate production by introducing heterologous lactate dehydrogenase^23^,^24^,^25^.

**Figure 3:**
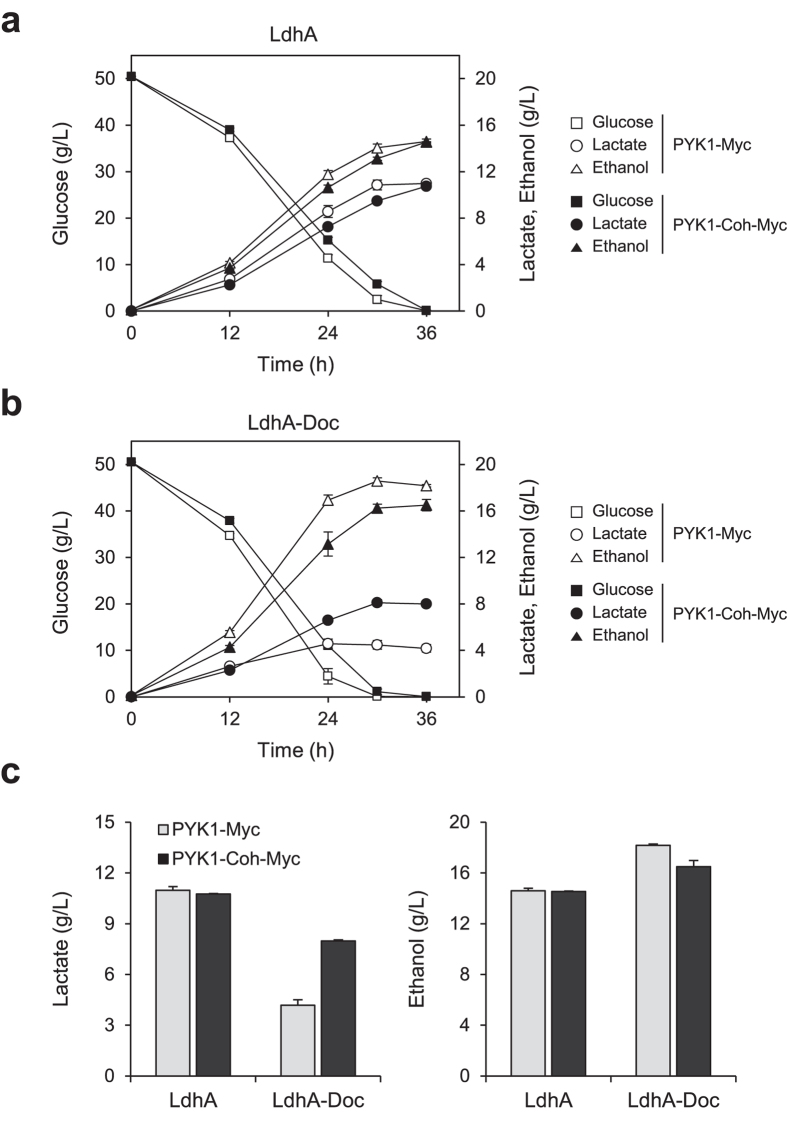
The effect of substrate channeling on lactate production. Fermentation profiles of PYK1-Myc (open symbol) and PYK1-Coh-Myc (closed symbol) strains, harboring (**a**) p415GPD-ldhA or (**b**) p415GPD-ldhA_Doc_. (**c**) The bar graphs show the production titers of lactate (left) and ethanol (right) after 36 h fermentation. Cells were grown in SC-Leu media containing 50 g/L glucose. Error bars indicate standard deviations of three independent experiments.

In this study, to produce lactate from pyruvate, lactate dehydrogenase LdhA from *Leuconostoc mesenteroides subsp. mesenteroides* ATCC 8293 was introduced into *S. cerevisiae*. This LdhA is specific for the production of D-lactate isomer^26^. First, we verified the effect of introducing native LdhA into PYK1-Myc or PYK1-Coh-Myc strain. Although the rates of glucose consumption and lactate production in PYK1-Coh-Myc strain were slightly lower than those of PYK1-Myc strain, there were no significant differences in the final production titers of lactate and ethanol between these two strains (Fig. 3a). Next, we investigated the substrate channeling effect on pyruvate distribution between lactate and ethanol by expressing dockerin-tagged LdhA (LdhA-Doc) in PYK1-Myc or PYK1-Coh-Myc strain (Fig. 3b). The PYK1-Myc control strain harboring p415GPD-ldhA_Doc_ produced 4.2 g/L lactate and 18.2 g/L ethanol after 36 h cultivation in SC-Leu medium containing 50 g/L glucose. On the other hand, PYK1-Coh-Myc strain harboring p415GPD-ldhA_Doc_ exhibited a 91% increase in lactate production (8.0 g/L) and a 9% decrease in ethanol production (16.5 g/L) compared with the control strain, demonstrating a successful substrate channeling effect between Pyk1 and LdhA (Fig. 3b). However, unfortunately, the final titer and yield of lactate decreased in both strains expressing LdhA-Doc compared with those in the strains expressing native LdhA (Fig. 3c). These results suggest that dockerin tagging might reduce the catalytic activity of LdhA, which limits the utilization of our substrate channeling strategy to improve lactate production. To confirm this, we determined the effect of dockerin tagging on the lactate dehydrogenase activity with purified enzymes, His_6_-LdhA and His_6_-LdhA-Doc. As expected, the specific activity of His_6_-LdhA-Doc (11.6 U/nmol) was lower than that of His_6_-LdhA (27.9 U/nmol) by more than two-fold.

### 2,3-Butanediol production using the pyruvate channeling system

The effect of dockerin tagging on enzymatic activity might be different depending on enzymes. Therefore, to further confirm the effect of metabolite channeling for pyruvate redistribution, we attempted to use PYK1-Coh-Myc strain to produce 2,3-butanediol (Fig. 1). 2,3-Butanediol is a valuable platform chemical used in extensive industrial applications such as a liquid fuel, solvent, and monomer of synthetic rubber. 2,3-Butanediol production from pyruvate using heterologous acetoin production pathway in *S. cerevisiae* has been described previously^27^. In our previous study to verify substrate channeling using a protein scaffold, three enzymes converting pyruvate to 2,3-butanediol including α-acetolactate synthase (AlsS) and α-acetolactate decarboxylase (AlsD) from *B. subtilis* and endogenous 2,3-butanediol dehydrogenase (Bdh1), were expressed as dockerin-fused proteins, and then assembled onto a scaffold containing multiple cohesin domains^21^. Because present study aimed to demonstrate the effect of metabolite channeling on the flux partitioning at a metabolic branch point, we focused on AlsS, a pyruvate converting enzyme. Therefore, AlsS was expressed as dockerin-fusion enzyme (AlsS-Doc) that enables its recruitment to Pyk1 via cohesin-dockerin interaction (Fig. 1).

**Figure 4:**
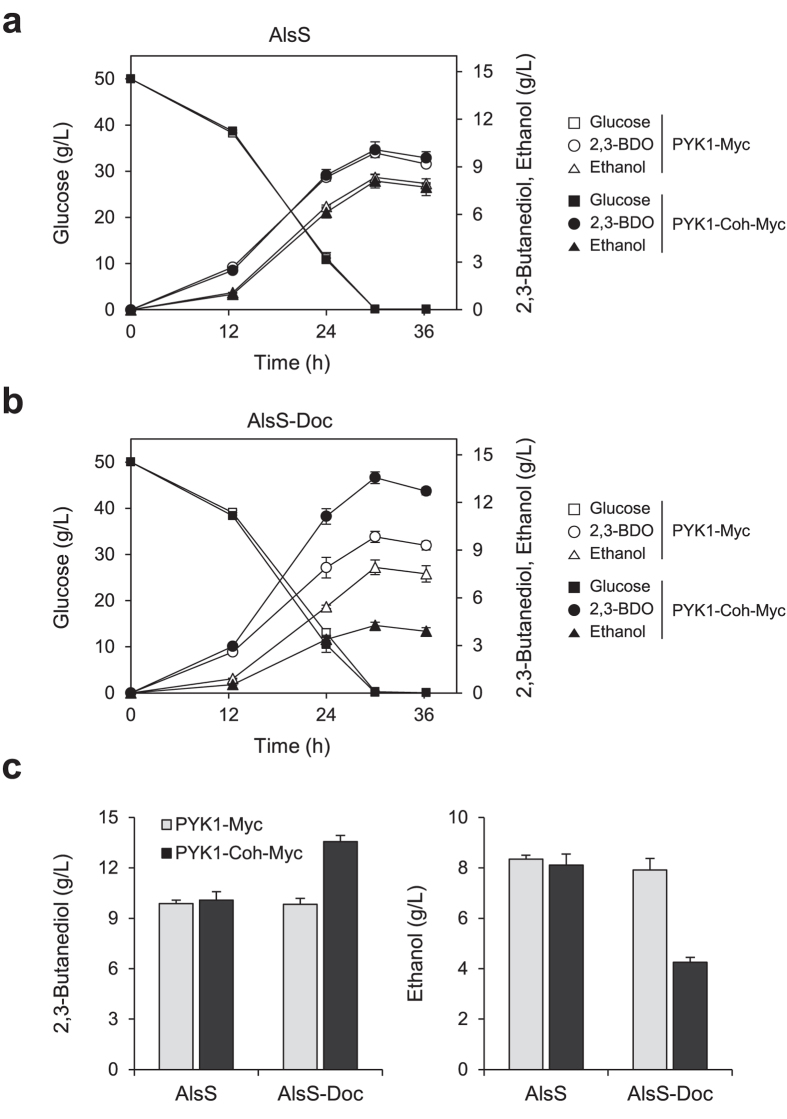
The effect of substrate channeling on 2,3-butanediol (2,3-BDO) production. Fermentation profiles of PYK1-Myc (open symbol) and PYK1-Coh-Myc (closed symbol) strains, both harboring (**a**) p413-SDB or (**b**) p413-S_Doc_DB. (**c**) The bar graphs show the production titers of 2,3-butanediol (left) and ethanol (right) after 30 h fermentation. Cells were grown in SC-His media containing 50 g/L glucose. Error bars indicate standard deviations of three independent experiments.

We tested the effect of introducing native AlsS together with AlsD and Bdh1 into PYK1-Myc or PYK1-Coh-Myc strain. As shown in Fig. 4a, there were no significant differences between these two strains, including glucose consumption rate and production of metabolites. After 30 h fermentation, PYK1-Myc and PYK1-Coh-Myc strains overexpressing native AlsS, AlsD, and Bdh1 from p413-SDB plasmid produced almost the same amounts of 2,3-butanediol (9.9 g/L and 10.1 g/L, respectively) and ethanol (8.3 g/L and 8.1 g/L, respectively) (Fig. 4a). These metabolite profiles during fermentation were also maintained in PYK1-Myc strain overexpressing AlsS-Doc, AlsD, and Bdh1 from p413-S_Doc_DB, which produced 9.8 g/L 2,3-butanediol and 7.9 g/L ethanol after 30 h, suggesting that dockerin tagging does not affect AlsS activity (Fig. 4b). In contrast, PYK1-Coh-Myc strain overexpressing AlsS-Doc, AlsD, and Bdh1 produced 13.6 g/L 2,3-butanediol, which indicates about 38% increase compared with that produced in PYK1-Myc strain. Consistent with this increase in 2,3-butanediol titer, ethanol production decreased by 46% (4.3 g/L after 30 h fermentation) (Fig. 4b,c). These results demonstrate that the pyruvate flux was successfully redistributed from ethanol toward 2,3-butanediol through the spatial organization of Pyk1 and AlsS.

## Discussion

As a proof of concept study, we investigated the effect of metabolite channeling on the flux partitioning at a metabolic branch point, focusing on pyruvate, a key intermediate of the central metabolism in *S. cerevisiae*. Especially, *S. cerevisiae* favors ethanol production from pyruvate in the presence of fermentable carbon sources even under aerobic conditions. Therefore, redirecting pyruvate flux to desired pathways in competition with ethanol production pathway is essential to produce pyruvate-derived chemicals. We constructed PYK1-Coh-Myc strain as a platform strain for pyruvate channeling and overexpressed pyruvate-converting enzymes with dockerin fusion. In the case of both lactate production and 2,3-butanediol production, pyruvate flux toward the target products was significantly enhanced, successfully competing with the native central metabolic pathway toward ethanol production even without genetic attenuation of the ethanol pathway enzymes such as pyruvate decarboxylase (Pdc) or alcohol dehydrogenase (Adh). As a result, it has been demonstrated that spatial organization of enzymes located at metabolic branch point could provide competitive advantages over the other pathways, within the frame of metabolite channeling effect.

Although cohesin tagging to Pyk1 and dockerin tagging to AlsS did not exert noticeable effects on enzyme activities, LdhA activity was severely reduced by dockerin tagging. Such a tagging-dependent inactivation of the protein function is one of the limitations of using substrate channeling strategies based on domain tagging. This problem might be solved by trying different protein-protein interaction domains or different tagging sites and linkers, which minimize the perturbation of the enzyme activity. The PYK1-Coh-Myc platform strain could also be applied to metabolic engineering strategies involving other pyruvate-converting enzymes whose activities are not largely diminished by dockerin tagging.

## Methods

### Strains and media

Yeast strains used in this study are listed in Table 1. *S. cerevisiae* CEN.PK2-1C was used as a parental strain. PYK1-Myc and PYK1-Coh-Myc strains were constructed by PCR-mediated homologous recombination based on Cre/*loxP* recombination system^28^. The integration cassettes were obtained by PCR amplification from pUG27-Myc and pUG27-Coh-Myc as templates, using a primer pair of 5′-*CGGTGCTGGTCACTCCAACACTTTGCAAGTCTCTACCGTT* GGTGGTTCTGGTGCTAGT-3′ and 5′-*TTCAAAAAAATAATATCTTCATTCAATCATGATTCTTTTT* GCATAGGCCACTAGTGGATC-3′ (sequences for homologous recombination were shown in italics). After confirmation of the correct integration of the cassette at the 3′ end of *PYK1* target gene locus through PCR analysis using the confirmation primers (5′-TGTCCACTTCCGGTACCACC-3′ and 5′-ATGCAACACCTCATCGTT-3′), the marker gene was removed by transformation of Cre recombinase-expression vector, p414GPD-Cre.

Yeast cells were cultured in YPD medium (10 g/L yeast extract, 20 g/L bacto-peptone, and 20 g/L glucose) or in synthetic complete (SC) medium (6.7 g/L yeast nitrogen base without amino acids and 1.4 g/L amino acids dropout mixture lacking His, Trp, Leu, and Ura) supplemented with auxotrophic amino acids as required and 20 or 50 g/L glucose.

### Plasmid construction

Plasmids used in this study are listed in Table 1. To construct C-terminal tagging vector, pUG27 plasmid containing *loxP*-*his5*^+^-*loxP* cassette was used as a starting plasmid. The DNA fragment sequentially consisting of linker sequence (GGSG), Myc epitope tag, and *CYC1* terminator (T_*CYC1*_) was cloned into HindIII and SalI sites of pUG27, resulting in pUG27-Myc plasmid. The cohesin domain from *Clostridium thermocellum* CipA was obtained by PCR amplification using p416GPD-GST-[Coh]_2_ as template^21^. The PCR product was cloned into the BamHI site located between linker sequence and Myc epitope tag in pUG27-Myc plasmid, resulting in pUG27-Coh-Myc. In order to omit the procedure of galactose induction for the expression of Cre recombinase, the DNA fragment encoding *cre* recombinase was obtained by cutting the pSH63 plasmid with SpeI and XhoI and cloned into p414GPD plasmid, resulting in p414GPD-Cre. HA-tagged *BDH1* was amplified by using PCR primer containing HA tag sequence and cloned into p415GPD, resulting in p415GPD-HA-BDH1. A plasmid p415GPD-HA-BDH1-Doc, expressing N-terminal HA-tagged Bdh1 with C-terminal dockerin fusion, was previously reported^21^. p415GPD-ldhA^29^, containing *ldhA* (LEUM_1756, D-lactate dehydrogenase) gene from *L. mesenteroides* ATCC 8293, was used for lactate production. The PCR product encoding *ldhA* without stop codon was prepared by PCR amplification and cloned into p415GPD-Doc^21^, resulting in p415GPD-ldhA_Doc_. For the expression of N-terminal His-tagged proteins, *E. coli* expression vector pET28b was used. The DNA fragment encoding *ldhA* or dockerin-tagged *ldhA* (*ldhA*-Doc) was obtained by cutting the p415GPD-ldhA or p415GPD-ldhA_Doc_ with SpeI and XhoI, and cloned into NheI and XhoI sites of pET28b plasmid, resulting in pET28b-ldhA or pET28b-His-ldhA-Doc, respectively. The construction of p413-SDB, a multigene-expression vector for 2,3-butanediol pathway, was described previously^30^. The DNA fragment encoding dockerin-tagged *alsS* was obtained from the p413GPD-alsS-Doc plasmid^21^ and cloned into p414_P_*TDH3*_/T_*PYK1*_, resulting in p414_P_*TDH3*_-*alsS-*Doc-T_*PYK1*_. [*alsS*-Doc]-expression cassettes (P_*TDH3*_-*alsS-*Doc-T_*PYK1*_) flanked by MluI and NotI sites was obtained using the universal primers^30^ and cloned into AscI and NotI sites of p413-DB, resulting in p413-S_Doc_DB.

### Co-immunoprecipitation

The PYK1-Coh-Myc strain harboring p415GPD-HA-BDH1 or p415GPD-HA-BDH1-Doc was grown in SC-Leu medium containing 20 g/L glucose and harvested at early log phase. The cells were resuspended in 250 μl of IP150 buffer [50 mM Tris-HCl (pH 7.4), 150 mM NaCl, 2 mM MgCl_2_, and 0.1% NP-40)] supplemented with 0.1% protease inhibitor cocktail (Calbiochem) and 1 mM PMSF. After an equal volume of acid-washed glass beads was added to each sample, cells were lysed by vortexing 10 times for 1 min at full speed with 1 min intervals on ice. Whole-yeast lysates (700 μg of total protein) were incubated with 1 μg c-Myc antibody (Santa Cruz Biotechnology) for 3 h at 4 °C followed by addition of 15 μL protein G PLUS-Agarose beads (Santa Cruz Biotechnology). After further incubation for 90 min at 4 °C, samples were washed three times with IP150 buffer and the bound proteins were eluted by boiling in sample buffer for 10 min. Samples were resolved by SDS-PAGE and proteins were detected by immunoblotting with the use of antibodies against Myc (Santa Cruz Biotechnology) and HA epitope tag (Roche Life Science).

### Protein expression and purification

*Escherichia coli* Rogetta gami2 (DE3) pLysS was used as a host strain to express N-terminal His-tagged proteins. The bacteria harboring pET28b-His-ldhA or pET28b-His-ldhA_Doc_ were grown to OD_600_ of 0.8 in LB medium supplemented with 50 μg/mL kanamycin at 37 °C, and induced with 1 mM isopropyl 1-thio-β-D-galactopyranoside (IPTG) at 30 °C for 4 h with shaking at 170 rpm. Cells were harvested, resuspended in binding buffer [20 mM Tris-HCl (pH 8.0), 500 mM NaCl, and 30 mM imidazole] supplemented with 0.1% protease inhibitor cocktail (Calbiochem) and 1 mM phenylmethylsulfonyl fluoride (PMSF), and lysed by sonication on ice for a total pulsing time of 10 min (pulse on for 15 s and off for 15 s). The cell lysates were centrifuged at 8,000 × *g* for 15 min at 4 °C and the supernatant samples were incubated with HisPur Ni-NTA resin (Thermo Scientific) for 2 h at 4 °C. After the Ni-NTA resin was washed twice with wash buffer [20 mM Tris-HCl (pH 8.0), 500 mM NaCl, and 45 mM imidazole], enzymes were eluted with elution buffer [20 mM Tris-HCl (pH 8.0), 500 mM NaCl, and 500 mM imidazole]. The expression and purity of the enzymes were confirmed by sodium dodecyl sulfate-polyacrylamide gel electrophoresis (SDS-PAGE) and the concentrations of purified enzymes were determined by the Bradford assay (Bio-Rad) with a bovine serum albumin standard.

### Lactate dehydrogenase activity assay

The enzyme activity of lactate dehydrogenase was determined as previously described^31^. Briefly, reduction of pyruvate to lactate was monitored by measuring the oxidation rate of NADH with a UV spectrophotometer at 340 nm. The enzyme activity was examined using 50 mM Tris-HCl (pH 7.0) buffer solution containing 10 mM sodium pyruvate and 0.2 mM β-NADH. One unit (U) of enzyme activity was defined as the amount that catalyzed the oxidation of 1 μmol of NADH per minute at pH 7.0.

### Culture conditions

For the production of lactate, yeast cells harboring p415GPD-ldhA or p415GPD-ldhA_Doc_ were pre-cultured in SC-Leu medium containing 20 g/L glucose and diluted to OD_600_ of 0.3 in 7.5 mL of SC-Leu medium containing 50 g/L glucose in a 50 mL conical tube at 30 °C with shaking at 170 rpm. For the production of 2,3-butanediol, yeast cells harboring p413-SDB or p413-S_Doc_DB were cultured in the same condition of lactate production in SC-His medium instead of SC-Leu medium.

### Analytical methods

The concentration of metabolites in the culture medium was determined by high performance liquid chromatography (HPLC) with BioRad Aminex HPX-87H column as previously described^32^. Briefly, 800 μL of culture supernatants were collected and filtered through a 0.22 μm syringe filter. Analysis was performed with 5 mM H_2_SO_4_ as a mobile phase at a flow rate of 0.6 mL/min and refractive index (RI) detector. The column temperature and detector temperature were maintained at 60 °C and 35 °C, respectively.

## Additional Information

**How to cite this article**: Kim, S. *et al*. Redirection of pyruvate flux toward desired metabolic pathways through substrate channeling between pyruvate kinase and pyruvate-converting enzymes in *Saccharomyces cerevisiae. Sci. Rep.* **6**, 24145; doi: 10.1038/srep24145 (2016).

## Figures and Tables

**Table 1:** Strains and plasmids used in this study.

Name	Description	Reference
Strains		
CEN.PK2-1C	*MAT***a** *ura3-52 trp1-289 leu2-3,112 his3Δ1 MAL2-8C SUC2*	EUROSCARF
PYK1-Myc	CEN.PK2-1C *PYK1*-Myc	This study
PYK1-Coh-Myc	CEN.PK2-1C *PYK1*-Coh-Myc	This study
Plasmids		
pUG27	Plasmid containing *loxP*-*his5*^+^-*loxP* deletion cassette	EUROSCARF
pSH63	CEN/ARS, *TRP1*, P_*GAL1*_-*cre*-T_*CYC1*_	EUROSCARF
pET28b	His_6_-tagged protein expression plasmid, Kan^R^	Novagen
p414GPD	CEN/ARS, *TRP1*, P_*TDH3*_, T_*CYC1*_	33
p415GPD	CEN/ARS, *LEU2*, P_*TDH3*_, T_*CYC1*_	33
p416GPD-GST-[Coh]_2_	CEN/ARS, *URA3*, P_*TDH3*_-[GST-(Coh)_2_-Doc]-T_*CYC1*_	21
pUG27-Myc	Plasmid containing Myc-[*loxP*-*his5*^+^-*loxP*] integration cassette	This study
pUG27-Coh-Myc	Plasmid containing Cohesin-Myc-[*loxP*-*his5*^+^-*loxP*] integration cassette	This study
p414GPD-Cre	CEN/ARS, *TRP1*, P_*TDH3*_-*cre*-T_*CYC1*_	This study
p415GPD-HA-BDH1	CEN/ARS, *LEU2*, P_*TDH3*_-[HA-*BDH1*]-T_*CYC1*_	This study
p415GPD-HA-BDH1-Doc	CEN/ARS, *LEU2*, P_*TDH3*_-[HA-*BDH1*-Doc]-T_*CYC1*_	21
p415GPD-Doc	CEN/ARS, *LEU2*, P_*TDH3*_-Doc-T_*CYC1*_ (Dockerin tagging plasmid)	21
p415GPD-ldhA	CEN/ARS, *LEU2*, P_*TDH3*_-*ldhA*-T_*CYC1*_	29
p415GPD-ldhA_Doc_	CEN/ARS, *LEU2*, P_*TDH3*_-[*ldhA*-Doc]-T_*CYC1*_	This study
pET28b-His-ldhA	His_6_-ldhA expression plasmid	This study
pET28b-His-ldhA_Doc_	His_6_-ldhA-Doc expression plasmid	This study
p413GPD-alsS-Doc	CEN/ARS, *HIS3*, P_*TDH3*_-[*alsS*-Doc]-T_*CYC1*_	21
p414_P_*TDH3*_/T_*PYK1*_	CEN/ARS, *TRP1*, P_*TDH3*_, T_*PYK1*_	30
p414_P_*TDH3*_-alsS-Doc-T_*PYK1*_	CEN/ARS, *TRP1*, P_*TDH3*_-[alsS-Doc]-T_*PYK1*_	This study
p413-DB	CEN/ARS, *HIS3*, P_*TEF1*_-*alsD*-T_*PYK1*_, P_*TPI1*_-*BDH1*-T_*TPI1*_	30
p413-SDB	CEN/ARS, *HIS3*, P_*TDH3*_-*alsS*-T_*PYK1*_, P_*TEF1*_-*alsD*-T_*PYK1*_, P_*TPI1*_-*BDH1*-T_*TPI1*_	30
p413-S_Doc_DB	CEN/ARS, *HIS3*, P_*TDH3*_-[*alsS*-Doc]-T_*PYK1*_, P_*TEF1*_-*alsD*-T_*PYK1*_, P_*TPI1*_-*BDH1*-T_*TPI1*_	This study
